# Antifibrinolytic drugs for acute traumatic injury

**DOI:** 10.1590/S1516-31802011000500014

**Published:** 2011-09-01

## Abstract

**BACKGROUND:**

Uncontrolled bleeding is an important cause of death in trauma victims. Antifibrinolytic treatment has been shown to reduce blood loss following surgery and may also be effective in reducing blood loss following trauma.

**OBJECTIVE:**

To quantify the effect of antifibrinolytic drugs in reducing blood loss, transfusion requirement and mortality after acute traumatic injury.

**CRITERIA FOR CONSIDERING STUDIES FOR THIS REVIEW:**

We searched the Cochrane Injuries Group's Specialised Register, CENTRAL, MEDLINE, PubMed, EMBASE, Science Citation Index, National Research Register, Zetoc, SIGLE, Global Health, LILACS, and Current Controlled Trials. The Cochrane Injuries Group Specialised Register, CENTRAL, MEDLINE and EMBASE searches were updated in July 2010.

**SELECTION CRITERIA:**

We included all randomised controlled trials of antifibrinolytic agents (aprotinin, tranexamic acid [TXA] and epsilon-aminocaproic acid) following acute traumatic injury.

**DATA COLLECTION AND ANALYSIS:**

The titles and abstracts identified in the electronic searches were screened by two independent authors to identify studies that had the potential to meet the inclusion criteria. The full reports of all such studies were obtained. From the results of the screened electronic searches, bibliographic searches, and contacts with experts, two authors independently selected trials meeting the inclusion criteria, with any disagreements resolved by consensus.

**MAIN RESULTS:**

Four trials met the inclusion criteria. Two trials with a combined total of 20,451 patients assessed the effects of TXA on mortality; TXA reduced the risk of death by 10% (RR = 0.90, 95% CI 0.85 to 0.97; P = 0.0035). Data from one trial involving 20,211 patients found that TXA reduced the risk of death due to bleeding by 15% (RR = 0.85, 95% CI 0.76 to 0.96; P = 0.0077). There was no evidence that TXA increased the risk of vascular occlusive events or need for surgical intervention. There was no substantial difference in the receipt of blood transfusion between the TXA and placebo groups. The two trials of aprotinin provided no reliable data.

**AUTHORS’ CONCLUSIONS:**

TXA safely reduces mortality in bleeding trauma patients without increasing the risk of adverse events. Further trials are needed to determine the effects of TXA in patients with isolated traumatic brain injury.

**Professor of Surgery, Faculdade de Medicina da Universidade de São Paulo (FMUSP); and Specialist in Intensive Medicine, Associação Médica Brasileira (AMB) and Federación Panamericana e Ibérica de Sociedades de Medicina Crítica y Terapia Intensiva (FEPIMCTI).**

**This is the abstract of a Cochrane Review published in the Cochrane Database of Systematic Reviews (CDSR) 2011, Issue, 1, DOI:** 10.1002/14651858.CD004896.pub3 (www.thecochranelibrary.com). For full citation and authors details see reference 1.

**DOI:** 10.1002/14651858.CD004896.pub3

**For Latin America and the Caribbean, the full text is freely available from:** http://cochrane.bvsalud.org/cochrane/show.php?db=reviews&mfn=2894&id=CD004896&lang=en&dblang=&lib=COC&print=yes.

## COMMENTS

Not much data on the use of antifibrinolytic agents following acute traumatic injury is available. This Cochrane review^1^ aimed to prove that antifibrinolytic treatment reduces blood loss following trauma and reduces the mortality rate. The review concludes that this is true, but this conclusion is based on a single well randomized study, CRASH-2 2000. The use of tranexamic acid (TXA) reduced the mortality rate with no apparent increased risk of vascular occlusive events.

This evidence will tend to promote wider use of TXA, in relation to aprotinin and epsilon-aminocaproic acid, over the first hours of attending to cases of bleeding trauma. New counter-checks can then be published, thereby increasing our knowledge and constituting new evidence.

## References

[1] Roberts I, Shakur H, Ker K, Coats T, CRASH-2 Trial collaborators (2011). Antifibrinolytic drugs for acute traumatic injury. Cochrane Database Syst Rev.

